# Mediating role of resilience in the relationship between mindfulness and mental health

**DOI:** 10.3389/fpsyg.2025.1570016

**Published:** 2025-07-15

**Authors:** Nishit Kumar Sinha

**Affiliations:** Department of Organizational Behaviour and Human Resources Management, Indian Institute of Management, Indore, India

**Keywords:** mindfulness, resilience, mental health, emotional well-being, psychological well-being, social well-being

## Abstract

Past research has established the beneficial effects of mindfulness on mental health. The present study attempts to extend the previous literature by examining the potential mediating role of resilience between mindfulness and mental health relationships. A survey questionnaire comprising the Cognitive and Affective Mindfulness Scale-Revised (CAMS-R), Wagnild and Young Brief Resilience scale, and Mental Health Continuum–Short Form (MHC-SF) was administered to 431 respondents from India. Confirmatory factor analysis confirmed the tripartite structure of MHC–SF, developed by to facilitate mental health assessment (including its three components: emotional, psychological, and social), in the Indian context. The study outcomes revealed that mindfulness was significantly associated with mental health (β = 0.472, *p* < 0.001) and its three dimensions: emotional (β = 0.405, *p* < 0.001), psychological (β = 0.480, *p* < 0.001), and social well-being (β = 0.296, *p* < 0.001). Resilience mediated the relationship between mindfulness and mental health, and its dimensions. The study findings provide information regarding the previously unknown resilience process through which mindfulness exerts its beneficial effects on mental health.

## 1 Introduction

Mental health is not merely the absence of mental disease. World Health Organization (WHO) observed mental health as a positive state, and defines it as “Mental health is a state of well-being in which every individual realizes his or her own potential, can cope with the normal stresses of life, can work productively and fruitfully, and is able to make a contribution to her or his community” (World Health Organization, 2004, p.12). Such a conceptualization of mental health is also consistent with the general definition of health as a state comprising of the presence of well-being, and not only the absence of any disease or disability (World Federation for Mental Health, 1948), which echoes well with the longstanding well-being traditions of hedonic well-being (concerned with the feelings of happiness) and the eudaimonic well-being (focussing upon optimal functioning in individual and social life) (Keyes, 1998; Waterman, 1993). Evidently, mental health comprises three core components: the presence of positive feelings (emotional well-being), functioning effectively in life (psychological well-being), and positive functioning in community life (social well-being). Because mental health is of critical importance in the overall functioning of individuals and societies (Barry, 2009), several recent studies have attempted to identify the dispositional antecedents, such as mindfulness, in promoting mental health (Duan, 2016; Shapiro et al., 2007).

Mindfulness is “being attentive to and aware of what is taking place in the present” (p. 822) (Brown and Ryan, 2003), characterized by non-judgmental awareness of the present and non-reactive attention to one’s own thoughts, sensations, feelings, and emotions (Kabat-Zinn, 2009). Previous studies have established that trait mindfulness is positively associated with multiple well-being conceptualizations. (e.g., Brown and Ryan, 2003; Schutte and Malouff, 2011). Various studies have also exhibited that mindfulness promotes psychological well-being (e.g., Brown and Ryan, 2003). There is preliminary evidence of the mediating relationship between mindfulness and different well-being conceptualizations. For example, the relationship between mindfulness and social well-being is explained through connectedness with nature (Howell et al., 2011). Similarly, mindfulness is associated with subjective well-being through mediators, including self-esteem and emotional intelligence (Pepping et al., 2013; Schutte and Malouff, 2011). Notwithstanding these studies, the mediating mechanisms allowing information related to the processes through which mindfulness affects several well-being forms are somewhat unsatisfactory (Bajaj and Pande, 2016). Concurrently, the mechanism through which mindfulness benefits positive mental health and its individual components (Keyes et al., 2008) is not fully understood yet. Specifically, the intrapsychic mechanism explaining the relationship between mindfulness and the individual components of mental health, viz, emotional well-being, psychological well-being and social well-being is yet to be explained.

Several mindfulness scholars (e.g., Luberto et al., 2014) have emphasized the need to identify the underlying mechanism through which mindfulness benefits several outcome variables. Baer (2015) proposed that several personal strengths may mediate the relationship between mindfulness and positive mental health. Because resilience is associated with various conceptualizations of well-being, including psychological well-being (Karreman and Vingerhoets, 2012), subjective well-being (Bajaj and Pande, 2016), and social competencies and connectedness (Camfield, 2012), we expect that mindfulness may convey its effects on the three individual components as well as on the overall mental health through resilience.

Resilience is a trait “that enables one to thrive in the face of adversity” (Connor and Davidson, 2003) through adaptability when coping with change and social problem-solving skills (Rutter, 1985). Thompson et al. (2011) found that mindful awareness and acceptance orientation toward experiences promotes psychological resilience by preventing ruminative and depressive thinking. Because moment-to-moment experiences characterize mindfulness, mindful individuals find it easier to break the habitual worrying and ruminative cycle (Verplanken and Fisher, 2014), maintaining a solution-oriented outlook even in the presence of unpleasant experiences (Hayes et al., 2011), thereby possessing greater resilient abilities. Resilience is expected to be more pronounced in mindful people because of their greater ability to be more creative and respond to tough circumstances without reacting in non-adaptive ways. Mindfulness present-centered awareness component provides greater access to consciousness, resulting in ‘healthy engagement’ with the negative affect (Chambers et al., 2009), through which mindful people simply observe and label the negative emotional state, instead of getting immersed in it. Relatedly, Lyons (1991) cited tolerance of negative affect and the ability to adapt as the key strategies responsible for advancing resilience. Basis these findings, there is preliminary evidence that mindfulness and resilience are positively associated (Sünbül and Güneri, 2019).

Resilient people possess a greater ability to maintain their psychological health by buffering adverse outcomes from difficult times (Connor and Davidson, 2003). Such people have a higher propensity to ask, “What am I going to do?” in the face of adversity, which initiates the resilient reintegration process, an introspective experience involving identifying, accessing, evaluating, and opting for suitable healing action (Richardson, 2002). The essence of such resilient reintegration lies in the enrichment of the protective factors and getting back to homeostasis to heal oneself (Richardson, 2002). There is strong evidence suggesting resilience to be positively associated with satisfaction with life and positive affect and negatively related to negative affect (Lü et al., 2014; Singh and Yu, 2010). Thus, resilience may act as an important resource for emotional well-being.

Individuals with higher resilience have a greater belief that they control their functioning, which acts as an enabler to cope with change and use a combination of problem-solving skills (Tusaie and Dyer, 2004). In a meta-analytic study, Lee et al. (2013) found resilience to be closely related to self-efficacy, a belief that one controls self-motivation, behavior, and what occurs in the environment (Bandura, 1997). Resilience is also construed as a protective process active in developing favorable judgments toward one’s self-worth (Lee et al., 2013). Because resilient people view change or stress as an opportunity and welcome new experiences through a positive reinterpretation strategy (Kobasa, 1979), they recognize not only the need for improvement in their behavior and self over the time but also have greater acceptance toward their past and positive attitude toward their persona (Sagone and De Caroli, 2014). Studies have suggested that resilient people are more likely to exhibit adaptive behavior, particularly in social functioning areas. Such resilient people are socially active and also tend to derive satisfaction with life from their ability to integrate well within society (Wagnild and Young, 1993). Literature supports the idea that resilience is positively associated with the eudaimonic paradigm of well-being, measured using psychological well-being and social well-being (Masten et al., 1999; Picardi et al., 2012; Ryff and Singer, 2000). Thus, we expect resilience to act as a motivator for psychological and social well-being.

Various studies have shown mindfulness to be significantly associated with overall mental health measured by MHC-SF and its dimensions EWB, PWB, and SWB (e.g., Bohlmeijer et al., 2011). Building upon the preceding rationale and existing literature exhibiting mindfulness as an antecedent to resilience and resilience positively correlated with mental health and its three components, namely, emotional, psychological, and social well-being, we expect that mindfulness exerts its salient benefits on overall positive mental health and its three components through resilience. In the presence of mindfulness, individuals are likely to be more resilient, thereby increasing the EWB, PWB, SWB, and overall mental health.

Mental Health Conceptualization and Hypotheses Development

Modern thinkers revived the idea behind happiness and brought forth questions such as “What is happiness?” and “How should it be measured?” (Baumeister et al., 2016; Buss, 2000). As there can be multiple interpretations of the possible meaning of happiness, a general understanding emerged to use a broader umbrella term called “well-being.” Building upon the thoughts of ancient philosophers, two schools of thought (related to well-being) emerged. The first thought equates well-being with the feeling of happiness (hedonic conceptualization), while the second one resonates with the idea of the realization of human potential that leads to positive functioning in day-to-day life (eudaimonic conceptualization).

The hedonic tradition of well-being comprises positive evaluations of life in emotional terms, which trickles down to the presence of positive affect as well as the absence of negative affect. The hedonic tradition thus focuses mainly on emotional aspects (Diener, 1984). Several researchers have often employed the term “emotional well-being” to specify the hedonic aspect of well-being (Waterman, 1993). A significant component of emotional well-being is feeling good (Massé et al., 1998), which refers to the affective component, as well as a cognitive component that relates to feelings of satisfaction with life or the perception that wants have been met (Veenhoven, 1994). Similarly, instead of focusing on only the affective component, Argyle (1987) stressed the importance of the cognitive component and defined it as a thoughtful appraisal of the quality of life. Building upon the contemporary literature, the notion of well-being comprising affective and cognitive components is supported by the conceptualization of subjective well-being by Diener (1984), who termed life satisfaction as “a global judgment that people make when they consider their life as a whole” (Diener, 1994).

At the same time, the eudaimonic tradition views well-being from a principally psychological perspective by coupling its meaning predominantly with the idea of self-fulfillment, expression, and the realization of the potential of an individual, and their ability to forge interpersonal relationships (Ryff and Keyes, 1995). The classical approach suggested by Aristotle revolved around the concept of “daimon” (Kraut, 2015). “Daimon” refers to “those potentialities of each person, the realization of which represents the greatest fulfillment in living of which each is capable” (Waterman, 1993). Thus, “daimon” represents an ideal, or an idea of perfection toward which one should strive, which gives meaning and direction to life (Waterman, 1993). An attempt to live in accordance with the “daimon,” to realize one’s full potential, leads to this approach of living one’s life—known as eudaimonia. Building upon, modern thinkers argued that certain aspects of realizing one’s full potential and positive functioning (e.g., working toward achieving one’s goals) might require a good amount of effort as well as discipline, which may be at odds with the pursuit of short-term happiness (Waterman, 1984). As discussed earlier, several philosophical discourses caution against happiness being the ultimate goal, and the idea that happiness is not an end in itself, but the byproduct of more noble pursuits (Mill, 1989). Such an idea is also supported by the conceptual model of psychological well-being, as proposed by Ryff (1989).

Going beyond that, Diener and Seligman (2004) proclaimed that “supportive and positive social relationships are critical to well-being,” and across society, the differences in individual well-being are frequently seen due to social relationships and not primarily because of income. Echoing the sentiment, the economist Helliwell (2003), proclaimed that individuals with the highest well-being are not those residing in the wealthiest and most developed countries but those in countries with strong institutions (such as effective social institutions) and strong feelings of mutual trust. Similarly, Putnam (2001) exhibited that communities with higher rates of social involvement, such as higher incidences of involvement in volunteer activities, memberships in clubs, had higher levels of well-being than communities with lower scores on these features. On similar lines, communities with a higher level of membership in non-work-related organizations had lower levels of suicide rates and higher levels of well-being (Helliwell, 2003). Subjective (Diener, 1984) and psychological well-being (Ryff, 1989) conceptualizations emphasized on measuring the private aspects of an individual’s life. This highlights the fact that individuals are embedded in social structures within multiple social relationships and face numerous opportunities as well as challenges while interacting with community members. These studies provide evidence that social involvement, social interactions, and societal connectedness within the community profoundly influence the level of well-being of the people. Thus, Larson (1992) put forward the idea that to report optimal functioning of the people, it is imperative to measure their social well-being. Building upon this narrative, Keyes (1998) established the social well-being model to examine and report on the quality of people’s social lives.

While psychological well-being is primarily described as a private phenomenon that centers on the challenges faced by people in their private sphere, social well-being conceptualization deals with the public phenomena that involve social tasks faced in people’s social sphere (Gallagher et al., 2009). Moving forward, Keyes et al. (2008) developed a brief questionnaire, “Mental Health Continuum-Short Form” (MHC-SF), suggesting a three-factor structure (emotional, psychological, and social) of well-being, derived from the 40-items long-form (MHC-LF) used in the Midlife in the United States (MIDUS) study (Keyes, 2002). This conceptualization of mental health as a combination of hedonic and eudaimonic (consisting of psychological and social dimensions) well-being has been cited by many researchers as a potent tool to report mental health conditions of community members (Joshanloo et al., 2013; Keyes, 2009; Lamers et al., 2011; Petrillo et al., 2015).

MHC-SF was created to parsimoniously and efficiently address the problem of mental health diagnosis. Notwithstanding the presence of some studies supporting the tripartite structure of mental health (emotional, psychological, and social well-being) in the United States (e.g., Gallagher et al., 2009)) and across cultures (Keyes, 2013), MHC-SF has not been widely used in the Indian context. With this background, the present study may contribute to the literature in the following way: (i) we expect to confirm the correlated three-factor structure of MHC-SF in the Indian context, (ii) the present study may provide additional support to the mental health literature by replicating the connections between mindfulness and the comprehensive conceptualization of mental health. Finally, (iii) through examining the previously unknown mediating role of resilience between mindfulness and individual components of MHC-SF and its dimensions (emotional, psychological, and social well-being) (Keyes et al., 2008) in an integrative manner, we expect to provide information regarding one possible process through which mindfulness benefits mental health.

Mindfulness is directly associated with higher levels of subjective well-being, comprising satisfaction with life, positive affect, and the absence of negative affect (Baer et al., 2008; Brown and Ryan, 2003). Mindful individuals find it easier to regulate their emotions (Shapiro et al., 2007), thus leading to the hypothesis that mindfulness is expected to positively associate with emotional well-being.

H1a: Mindfulness is positively associated with emotional well-being.

Mindfulness is related to lower levels of neuroticism (Brown and Ryan, 2003), lower levels of anxiety and depressive tendencies (Sinha et al., 2021a) and impulsive tendencies (Vihari et al., 2022). Mindfulness is also associated with higher autonomy, competence and relatedness – the essential needs as specified by self-determination (SDT; Deci and Ryan, 1980). Satisfaction with these needs provides the essential background for growth, health, and well-being, leading to a higher tendency to experience optimal functioning and fulfillment (Ryan and Deci, 2000).

H1b: Mindfulness is positively associated with psychological well-being.

Mindfulness promotes a non-judgmental attitude and higher awareness of environmental cues; mindful individuals do not exhibit reactive and automatic behavior even in difficult situations (Brown and Ryan, 2003). Thus, Mindful people are adept at social relationships, foster quality interpersonal relationships (Pratscher et al., 2018) and are expected to experience higher social well-being.

H1c: Mindfulness is positively associated with social well-being.

Self-regulation (of attention) is an essential benefit of mindfulness (Bishop et al., 2004), thus regulating the compulsive and impulsive behaviors. Such individuals are more guided by the intrinsic fulfilment needs (such as relatedness) than the extrinsic ones (such as wealth accumulation) (Kasser and Ryan, 1996). Studies have found that mindfulness promotes financial well-being, and such individuals do not have unhealthy obsessions about the future (Sinha et al., 2021b). Mindful individuals enjoy moment-to-moment experiences, thus bringing life to the present moment (Nilsson and Kazemi, 2016). Thus, the study expects mindfulness to be positively related to mental health.

H1d: Mindfulness is positively associated with positive mental health.

Previous studies have highlighted that in the presence of mindfulness, an individual is expected to exhibit greater resilience (e.g., Sünbül and Güneri, 2019). Further, resilience is found to be positively associated with emotional well-being (Bajaj et al., 2022). Thus, the following hypothesis:

H2a: Resilience mediates the relationship between mindfulness and emotional well-being.

Previous literature supports the idea that resilience promotes psychological well-being (Sayed et al., 2024). Thus, the study hypothesizes that resilience is expected to mediate the relationship between mindfulness and psychological well-being.

H2b: Resilience mediates the relationship between mindfulness and psychological well-being.

Resilient people are better equipped to develop and maintain a strong and positive social relationship (Nemeth and Olivier, 2017). Thus, the study expects resilience to mediate mindfulness and social well-being relationship.

H2c: Resilience mediates the relationship between mindfulness and social well-being.

Resilience acts as a self-defense mechanism to bounce back faster from adverse conditions and thus is found to be positively associated with positive mental health (Davydov et al., 2010). Thus, resilience is expected to mediate the mindfulness and mental health relationship.

H2d: Resilience mediates the relationship between mindfulness and positive mental health.

## 2 Materials and methods

### 2.1 Participants

We approached a convenience sample of 640 respondents from India. The data was collected using Google Form, from a large university in India. The university runs several undergraduate and postgraduate-level programs. The data was collected from the staff, faculty members, as well as the students. Out of which, 431 completed surveys were returned, yielding a 67% response rate. For the purpose of analysis, only the adult respondents were considered. Respondents (approximately 46% females) were aged 18–45 years (M_*age*_ = 23.85 years, S.D._*age*_ = 3.50). The respondents required for performing linear regression (effect size = 0.15, α = 0.01, power = 0.95, predictors = 2) were calculated to be 143 using GPower v3.1 (Faul et al., 2007). An effect size of 0.15 in social and personality psychology is considered modest (Richard et al., 2003). The participants were informed about the objective of the study and the voluntary nature of their participation. Participants were assured of responses being used only in an aggregated and anonymized manner.

### 2.2 Measures

#### 2.2.1 Trait mindfulness

Trait mindfulness was measured using the 12-items Cognitive and Affective Mindfulness Scale-Revised (CAMS–R) (Feldman et al., 2006). CAMS–R is designed to measure the four mindfulness dimensions: attention, awareness, present-focus, and non-judgment (e.g., “It’s easy for me to keep track of my thoughts and feelings”). These four dimensions have been emphasized as the core mindfulness themes (e.g., Bishop et al., 2004; Kabat-Zinn, 1990). The study chose CAMS-R (Feldman et al., 2006) over the Mindful Attention Awareness Scale (MAAS) (Brown and Ryan, 2003) for its unidimensional mindfulness measurement, not focusing upon attitudinal components of acceptance and non-judgment as the mindfulness components. Participants responded on a four-point scale from 1 (rarely/not at all) to 4 (almost always), after which the overall mindfulness score was computed. Higher score signifies higher mindfulness level.

#### 2.2.2 Resilience

Resilience was measured using the 6-item version of the resilience scale (e.g., reverse-coded item “I can get through difficult times because I have experienced difficulty before”) (Wagnild and Young, 1993) used in the psychological capital construct (Luthans et al., 2007). The study used the resilience scale (Wagnild and Young, 1993) over the Connor - Davidson Resilience Scale (CD - RISC) (Connor and Davidson, 2003) for two reasons: (i) studies have highlighted that CD-RISC failed to reproduce the originally envisaged five-factor structure highlighting that process of resilience differs in different cohorts (Cosco et al., 2016). (ii) Resilience scale (Wagnild and Young, 1993) has the “greatest number of validation studies” (Cosco et al., 2016, p.5), making it a prudent choice to measure resilience across different demographics. Respondents expressed their (dis) agreement using a six-point Likert scale (1 = strongly disagree, 6 = strongly agree) in which higher scores expressed higher resilience.

#### 2.2.3 Mental health

The study operationalized mental health continuum-short form (MHC-SF) (Keyes, 2009), which contains three items of emotional well-being, six items of psychological well-being, and five items of social well-being. The first dimension, emotional well-being (e.g., “During the past 30 days, how much of the time did you feel satisfied?”) measures a cognitive appraisal of satisfaction with life in general. The second dimension, psychological well-being (e.g., “During the past 30 days, how much of the time did you feel that your life has a sense of direction or meaning to it?”) reflects the extent to which individuals perceive themselves as having a meaningful life. The third dimension, social well-being, measures (e.g., “During the past 30 days, how much of the time did you feel that our society is becoming a better place for people?”) individuals’ evaluations of their public and social lives. Respondents expressed their opinion using a six-point Likert scale (0 = never, 5 = everyday).

## 3 Results

### 3.1 Preliminary tests of common method bias, reliability, validity, and correlation

We performed preliminary analyses (using SPSS v24.0) to test common method bias, reliability, and validity of the well-being scale (MHC-SF) used in our study. Common method bias exists when the variations in participant responses are a function of the instrument rather than their actual inclination. Despite some observed limitations of Harman’s one-factor test, such as influence of extraneous study design aspects, lack of quantification, and the issues of false negatives as well as false positives, especially in case of substantial bias cases, the study used it to assess common method bias (Podsakoff et al., 2012). Single-factor explained only 37.76% of the variance, which is lower than 50%, indicating a lower likelihood of common method bias. Principal component analysis (PCA) with varimax rotation used to identify the underlying factors conducted on the 15-items well-being scale resulted in a three-factor solution (eigenvalue greater than 1). Results indicated adequate KMO’s measure of sampling adequacy at.911. KMO values between 0.8–1.0 are statistically sufficient to conduct factor analysis (Cerny and Kaiser, 1977). Bartlett’s test of sphericity was significant (χ^2^= 2552, df = 91, *p*-value < 0.000). Also, Kaiser’s criterion and scree plot were employed as the criteria to determine the number of factors. The scree test (Bryman and Cramer, 2004), which plots the eigenvalues against the number of components, suggested three substantive factors. Table 1 provides an overview of the items and their loadings obtained after PCA.

**TABLE 1:** Principal component analysis with Varimax orthogonal rotation. Eigenvalues > 1. Factor loadings > 0.5. (*n* = 431). Rotation converged in 5 iterations.

Items	Well-being dimensions		
	Psychological well-being α = 0.82	Social well-being α = 0.85	Emotional well-being α = 0.76
I have something important to contribute to society		0.699	
I belonged to a community (like a social group, or neighborhood)		0.612	
Our society is becoming a better place, for all people		0.836	
People are basically good		0.719	
The way our society works makes sense to me		0.781	
I like most parts of my personality	0.726		
I am good at managing the responsibilities of my daily life	0.749		
I have warm and trusting relationships with others	0.549		
I have experiences that challenged me to grow and become a better person	0.732		
I am confident to think or express my ideas and opinions	0.772		
My life has a sense of direction or meaning to it	0.679		
Feeling of happiness			0.829
Feeling of satisfaction			0.767
Feeling of interest in life			0.645

**FIGURE 1:**
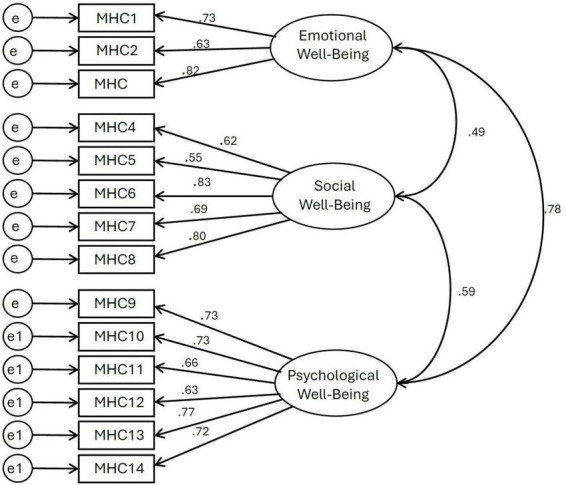
Confirmatory factor analysis of well-being three-dimensional structure.

Post that, we administered confirmatory factor analysis (CFA) to test the model fit of the proposed three-dimensional well-being model against the competing models. Hu and Bentler (1999) suggested several indices and their acceptable limit to evaluate the model fit: Comparative fit index (CFI) of 0.90 or above; root mean square of approximation (RMSEA) value less than 0.06 signifies a good fit. The Tucker-Lewis index (TLI) of 0.90 or above and χ^2^/df values between 1 and 3 indicate a good fit (Arbuckle and Wothke, 1999). The CFA results confirm that the three-dimensional structure, including emotional, psychological, and social well-being, is sufficiently fitted to the data (Figure 1) (χ^2^ (74) = 174.5, CFI = 0.964, TLI = 0.955, RMSEA = 0.053, and χ^2^/df = 2.22), and was considerably better than the one-factor model (χ^2^ (77) = 654.1, CFI = 0.769, TLI = 0.727, RMSEA = 0.132, and χ^2^/df = 8.49). We dropped two items–CAMS_2 (0.32 factor loading) and CAMS_6 (0.38 factor loading) - from the mindfulness scale owing to the poor factor loading. These two items are reverse-coded items in the scale. In the original measurement model of mindfulness with all the items, the following model fit indices were obtained: (χ^2^ (54) = 154.9, CFI = 0.815, TLI = 0.733, RMSEA = 0.066, and χ^2^/df = 2.87). In the accepted model, which is obtained after dropping two items, the following model fit indices were obtained: (χ^2^ (33) = 69.01, CFI = 0.931, TLI = 0.906, RMSEA = 0.050, and χ^2^/df = 2.09). In the accepted model, the standardized loadings for all the indicators on their respective latent variables were significant (p < 0.001), and had factor loadings above 0.5, satisfying Nunnally (1978) criteria of internal consistency. The model fit for resilience was adequate: (χ^2^ (8) = 15.4, CFI = 0.989, TLI = 0.980, RMSEA = 0.047, and χ^2^/df = 1.93).

Figure 1 presents the CFA results, including the correlations between each well-being dimension and factor loadings of items used in MHC–SF. All presented correlations are significant with *p* < 0.01.

Table 2 reports the bivariate correlations, descriptive statistics, and Cronbach’s alpha coefficients (using SPSS v24) of the study variables. Greater mindfulness was significantly associated with more emotional well-being (*r* = 0.405, *p* < 0.001), more social well-being (*r* = 0.296, *p* < 0.001), more psychological well-being (*r* = 0.480, *p* < 0.001) and positive mental health (*r* = 0.472, *p* < 0.001). Mindfulness exhibited a significant relationship with the proposed mediator resilience (*r* = 0.519, *p* < 0.001). Resilience was significantly associated with the three dimensions of well-being: emotional (*r* = 0.473, *p* < 0.001), social (*r* = 0.317, *p* < 0.001), psychological (*r* = 0.542, *p* < 0.001), and, positive mental health (*r* = 0.529, *p* < 0.001).

**TABLE 2:** Descriptive statistics, reliabilities, composite reliabilities (CR) and correlation matrix (*n* = 431).

Measures	Means	SD	α	CR	1	2	3	4	5	6
Mindfulness	2.83	0.47	0.69	0.80	1					
EWB	3.30	1.09	0.76	0.77	0.40**	1				
SWB	2.86	1.20	0.78	0.83	0.29**	0.42**	1			
PWB	3.46	1.01	0.85	0.86	0.48**	0.62**	0.51**	1		
Positive mental health	3.21	0.91	0.88	0.84	0.47**	0.75**	0.68**	0.78**	1	
Resilience	3.95	0.67	0.81	0.82	0.51**	0.47**	0.31**	0.54**	0.52**	1

**p-value < 0.01 (2-tailed). α = reliability estimates (Cronbach’s α) for the respective variables. EWB, emotional well-being; SWB, social well-being; PWB, psychological well-being.

### 3.2 Hypothesis testing

The normality and multicollinearity assumptions were evaluated before running the regression analysis. To examine the different determinants of positive mental health among the measures employed in the study, we computed the regression coefficients. First, we tested the impact of mindfulness on the three components of well-being. The regression coefficients exhibited that mindfulness was positively associated with EWB (β = 0.405, *p* < 0.001), SWB (β = 0.296, *p* < 0.001), PWB (β = 0.480, *p* < 0.001) and positive mental health (β = 0.472, *p* < 0.001), thus supporting H1a–H1d. Further, we tested the relationship between resilience and MHC-SF components. The regression model revealed that resilience was positively associated with EWB (β = 0.473, *p* < 0.001), SWB (β = 0.317, *p* < 0.001), PWB (β = 0.542, *p* = 0.015) and overall positive mental health (β = 0.529, *p* < 0.001). Mindfulness related positively with resilience (β = 0.519, *p* = 0.001). Thus, for further analysis, resilience was considered as the probable mediator between mindfulness and positive mental health and its three components.

To conduct the hypothesized mediation analysis, we used model 4 of the SPSS PROCESS macro (Preacher and Hayes, 2008). Following the method used by Schutte and Malouff (2011), we tested four separate mediation models, each including mindfulness as the exogenous variable and resilience as the mediator. For the four tests, the dependent variables were EWB, SWB, PWB, and positive mental health, respectively. Table 3 presents the mediation results of resilience on the relationship between mindfulness and EWB, SWB, PWB, and positive mental health.

**TABLE 3:** Mediating effect of resilience between mindfulness and EWB, SWB, PWB, and positive mental health. The value of R^2^ (measure of variance), as explained by mindfulness and resilience (mediator), is given in the last column.

Dependent variable with predictor and mediator	Multivariate analysis			R^2^ value
	B	T	*p*-value	
EWB				0.258
Mindfulness	0.218	4.48	< 0.001	
Resilience	0.359	7.37	< 0.001	
SWB				0.124
Mindfulness	0.179	3.38	< 0.001	
Resilience	0.224	4.23	< 0.001	
PWB				0.347
Mindfulness	0.271	5.94	< 0.001	
Resilience	0.401	8.77	< 0.001	
Positive mental health				0.333
Mindfulness	0.270	5.84	< 0.001	
Resilience	0.389	8.42	< 0.001	

Table 4 presents the direct, indirect, and total effects of mindfulness (exogenous variable) and resilience (mediator) on the well-being indices.

**TABLE 4:** Standardized direct, indirect, and total effect.

	On EWB	On SWB	On PWB	Mental health
Direct effect	0.0508	0.0460	0.0587	0.0392
Indirect effect	0.0434	0.0298	0.0450	0.0524
Total effect	0.0941	0.0758	0.1036	0.0917

Finally, the study used the bootstrapping method to verify mediation results (Preacher and Hayes, 2008). If the confidence intervals (CIs) contain zero, the mediating effect of the mediator becomes nonsignificant. The bootstrap method follows that if the sample is a true representative of the population, then the empirical distribution is expected to be the correct substitute for the population distribution. Examining the 95% bias-corrected CIs from 5000 bootstrapped samples (Table 5) supported the mediation effect of resilience between mindfulness and individual dimensions and the overall positive mental health relationship, thus supporting hypotheses 2a–2d. Thus, the findings suggest with a 95% confidence level that the true population parameter falls within the calculated intervals.

**TABLE 5:** Bootstrapping summary of the mediation results.

Hypotheses	Path	Indirect effect	Std. error	95% bias-corrected CI lower upper		Remarks
H2a	Mindfulness–resilience–EWB	0.186	0.028	0.131	0.243	Partial mediation
H2b	Mindfulness–resilience–SWB	0.116	0.030	0.055	0.173	Partial mediation
H2c	Mindfulness–resilience–PWB	0.208	0.026	0.158	0.260	Partial mediation
H2d	Mindfulness–resilience–Positive mental health	0.202	0.027	0.149	0.257	Partial mediation

## 4 Discussion

Economic uncertainties, social unrest, and volatility in the current situation have redrawn the focus of researchers on the antecedents of positive mental health. The absence of mental diseases does not imply the presence of positive mental health. Positive mental health is characterized by feeling good and functioning well in personal and social life. Under this backdrop, the present study embarked upon achieving the following objectives: (i) assessing the psychometric characteristics of the MHC–SF, a questionnaire designed for measuring positive mental health, in the Indian context, and (ii) identification of the malleable variables that may help cultivate positive mental health specifically focusing upon mindfulness and resilience. In doing so, we extended the previous findings by examining the potential mediating role of resilience in the impact of mindfulness on emotional well-being, psychological well-being and social well-being, and overall mental health.

The present study confirmed the three-factor model of the MHC-SF in the Indian context. All subscales of the MHC-SF and the overall scale exhibited good reliability. Various studies support the tripartite structure of mental health (emotional, social, and psychological well-being) across different geographical samples, including Italian (Petrillo et al., 2015), South African (Keyes et al., 2008), and Serbian (Joshanloo and Jovanoviæ, 2017). India is the 2nd most populous country, and mental health is a universal concern for individuals and societies. The present finding may provide support and pave the way for MHC-SF to be used in a broader context.

Consistent with the previous findings (e.g., Howell et al., 2011), we found that mindfulness positively associated with mental health and its dimensions (emotional, psychological, and social well-being), thus supporting H1a to H1d. Previous studies have found a similar positive association between mindfulness and mental health (Duan, 2016; Shonin et al., 2014), though, as stated earlier, in the present study, mental health conceptualization was more comprehensive, comprising a tripartite structure of well-being. The present study outcomes revealed that mindfulness significantly related with resilience, which is in sync with previous findings (Bajaj and Pande, 2016; Sünbül and Güneri, 2019). Although some studies have examined the role of mediators on the relationship between mindfulness and some well-being conceptualizations, to our knowledge, the present work is the first to investigate the mediating role of resilience between mindfulness and overall mental health, as measured by MHC-SF, and its factors in an integrated manner.

The present study findings empirically demonstrated that resilience may mediate the relationship between mindfulness and overall mental health and between mindfulness and the three components of mental health (EWB, PWB, SWB), thus supporting H2a–H2d. The underlying theoretical mechanism lies with the fact that the aim of mindfulness is not to suppress the affective feelings, instead, it intends to alter how present-moment experiences are interpreted. The ability to mindfully observe the affective experiences as mere mental events (Papies et al., 2015) is expected to provide emotional balance to recover from a misfortune faster (Davidson and Begley, 2013). Mindful awareness and acceptance aspects encourage prosocial peer relationships and develop effective coping strategies (Weinstein et al., 2009). Such mindful mechanisms may facilitate the development of greater resilience (Masten and Reed, 2002).

Similar to mindfulness orientation, resilience does not promote stress avoidance but instead encourages facing stress and other adverse experiences through attaching appropriate meaning to them and by using the appropriate degree of control and mastery (Rutter, 1985). As an individual’s response to any stressor is greatly influenced by the appraisal of the stimulus, and the perceived capability to cope with it, resilient individuals’ ability to adapt and act within the range of problem-solving skills helps them to deal with adverse situations positively. Such people hold the belief that it is normal to meet and overcome challenges, and for them coping with stressful situations and adversities acts as a strengthening measure. These findings thus furnish evidence of the connection between mindfulness and resilience and between those two characteristics and emotional well-being.

Resilient people have a greater realization of the meaningfulness of life, that life has a purpose, and there is something for which to live even in the wake of misfortunes (Wagnild and Young, 1990). Such people possess emotional stamina, which helps them display courage and adaptability during adversity through a realistic assessment of the stressor and using available resources effectively (Caplan, 1990). Thus performed repeated mastery, despite adverse circumstances, acts as an enabler in confidently handling new experiences and managing the environmental factors as well as impending uncertainty. Although resilient people are guided by the perception that while some experiences are shared, each person’s life path has uniqueness and should be faced alone (Frankl, 1985; Wagnild and Young, 1993), they invest in developing secured relationships that foster resilience capability (Byrne et al., 1986). Such people tend to develop resourcefulness, social competence, and social intimacy (Kadner, 1989) through which they engage in seeking help while in need and reciprocally offer support to others. Uncovering of such findings provide ample support to the notion that resilience is positively associated with psychological and social well-being. These findings empirically support the idea that in the presence of mindfulness, a resilient person is expected to find it easier to have positive mental health as well as emotional, psychological, and social well-being. Thus, the study findings provide one possible mechanism in the form of resilience through which mindfulness significantly exerts its beneficial effects on mental health.

## 5 Limitations and future directions

The present study has several limitations. First, the data relied exclusively on self-report measures. Though the measures exhibited good reliability, and we attempted to address biases, responses may have a component of social desirability. Future studies may include multi-rater design or use multiple assessment methods for evaluations, which may lessen the influence of subjectivity on well-being assessment. The multi-rater design may be particularly more useful in cases of measuring the mindfulness score of the children, who themselves may not have a clear understanding of the survey instruments. Second, the study used a cross-sectional design which has its limitations in determining a causal relationship. While interpreting the mediation analysis results, the usual cautions related to cross-sectional data is suggested. Future research may incorporate longitudinal as well as experimental studies, which may furnish additional insights into relationships between mindfulness, resilience, and the outcome variable. Because mindfulness and well-being are broad constructs, future researchers are advised to examine the role of other mediating variables. Also, previous studies have cited the role of other personality traits (e.g., conscientiousness) on mental health. Future researchers are suggested to control the effects of these variables. Third, the study used a sample drawn from a particular geographical and socio-economic background, studying at a university in India. This might limit the generalizability of the study findings. Future studies might use a sample drawn from multiple backgrounds. Fourth, the study used the short 6-item resilience scale to evaluate the hypothesized relationship. Future studies might use other resilience scales such as the Connor-Davidson Resilience Scale.

## 6 Implications

The theoretical contribution of the present work lies in identifying the dispositional antecedents of mental health, measured by MHC-SF. In doing so, we also found support for the factor structure (emotional, psychological, and social) of MHC-SF in the Indian context and thereby broadened its applicability. The study results also resonate well with the postulates of Self-Determination Theory (SDT; Deci and Ryan, 1980). SDT attempts to offer a distinction between intrinsic and extrinsic motivations that explain an individual’s behavior, influencing their well-being (Brown and Ryan, 2003). The study supported the idea that because mindful individuals are more intrinsically motivated and exhibit more engaged behavior, they tend to experience greater benefits in the form of mental health. The study outcomes suggest critical implications for framing public policy. Organizations, societies, and countries are progressively acknowledging the importance of positive mental health among their people. The study outcomes demonstrated that mindfulness promotes individual dimensions, as well as overall positive mental health, which is an asset and a resource for society’s long-term social and economic prosperity (Barry, 2009). Studies have established that mindfulness is a trainable quality, and various physical and online-based mindfulness interventions are efficacious in enhancing people’s mindfulness level (Bailey et al., 2018; Brown and Ryan, 2003). Also, several findings caution that students are more susceptible to adverse mental health issues, which has led to severe concerns about an increased demand for student mental health services (Galante et al., 2018). The present study found that mindfulness fosters enhanced resilience, establishing a case for mindfulness-based resilience programs, which are innovative and cost-effective (Meiklejohn et al., 2012), to be inducted within the school-based curriculum. Such a program may act as a practical means to build personal resources acting as a buffer against several life stressors that may negatively influence mental health, including emotional, social and psychological health. The mindfulness training programs at schools can be categorized for different age groups, for example, 3 to 6-year-olds, 6 to 11-year-olds, and 11 to 14-year-olds. The 3 to 6-year-olds can be taught about concentration, compassion, bodily sensations, and awareness of oneself. It is important that such young kids are taught in in-person settings, and not in an online mode. In the second group, 6 to 11-year-olds can be taught to focus and redraw their attentional mechanisms, how the brain functions, different bodily and emotional states, and methods to moderate their reactivity to nurture themselves and others. The third group, 11 to 14-year-olds, can be taught about breathing and its relationship with attention and awareness, developing the skills related to thoughtless awareness and a non-judgmental attitude toward self and environmental cues.

## Data Availability

The raw data supporting the conclusions of this article will be made available by the authors, without undue reservation.
